# Viral Hepatitis Endemicity and Trends among an Asymptomatic Adult Population in Ho: A 5-Year Retrospective Study at the Ho Municipal Hospital, Ghana

**DOI:** 10.1155/2017/6174743

**Published:** 2017-11-06

**Authors:** Sylvester Yao Lokpo, James Osei-Yeboah, Gameli Kwame Norgbe, Patrick Kwasi Owiafe, Felix Ayroe, Francis Abeku Ussher, Mavis Popuelle Dakorah, John Gameli Deku, Nana Yaw Barimah Manaphraim, Emmanuel Akomanin Asiamah, Tibemponi Ntoni, Prince Senyo Kwasi Nyamadi, Edward Yiadom Boakye, Roseline Avorkliyah

**Affiliations:** ^1^Department of Medical Laboratory Sciences, School of Allied Health Sciences, University of Health and Allied Sciences, Ho, Ghana; ^2^School of Allied Health Sciences, University of Health and Allied Sciences, Ho, Ghana; ^3^Laboratory Department, Ho Municipal Hospital, Ghana Health Service, Ho, Volta Region, Ghana; ^4^Faculty of Health and Allied Sciences, Koforidua Technical University, Koforidua, Eastern Region, Ghana; ^5^Laboratory Department, St. Dominic Hospital, Akwatia, Eastern Region, Ghana

## Abstract

**Background:**

Using prospective blood donors as a proxy, this study was aimed at estimating the burden and five-year (2012–2016) trend of viral hepatitis (HBV and HCV) infection among asymptomatic adult population in Ho.

**Materials and Methods:**

A retrospective analysis was done on secondary data extracted from the hospital archives comprising 4,180 prospective blood donors from January 2012 to December 2016. Demographic variables included age and sex, as well as place of residence. Screening results of serum infectious markers (HBV and HCV) were obtained.

**Results:**

The prevalence of asymptomatic viral hepatitis (HBV and HCV) infection in the general adult population was 6.94% and 1.84%, respectively. Females recorded a higher burden of HBV and HCV (8.3% and 5.0%) compared to their male peers (6.8% and 1.4%). A significant age variation in HBV antigenaemia was seen with HBV seropositivity peaking among the younger population (less than 20 years' group) at 11.24% and troughed among the older population (above 50 years' group) at 0.92%.

**Conclusion:**

Asymptomatic viral hepatitis among adult population in the Ho Municipality is estimated at the intermediate to high endemicity level. Preventive measures to reduce the burden are urgently needed and should be targeted at the younger generation.

## 1. Introduction

Hepatitis B virus (HBV) and hepatitis C virus (HCV) infections are a significant public health problem globally and in developing countries [1]. Recent World Health Organisation (WHO) report indicates that nearly 257 million people are chronically living with HBV infection while an estimated 71 million persons are suffering from chronic HCV infection [2]. In Sub-Saharan Africa, about 8% of the population are chronic carriers of HBV, whereas HCV infection rate is reported to range from 2.1%–2.8% with the highest rate of 2.8% in the West African subregion [3, 4]. In Ghana, the estimated national prevalence of HBV and HCV as determined through systematic reviews were 12.3% and 3%, respectively [5, 6]. Prolonged and untreated viral hepatitis could lead to complications such as liver fibrosis, cirrhosis, end-stage liver disease, hepatocellular carcinoma (HCC), and mortality due to liver pathology [5]. Estimating the burden and trend of infectious pathogens among carrier asymptomatic populations who serve as reservoirs for the infectious agents holds immense epidemiological and preventive importance, especially in endemic areas like Ghana. However, the choice of appropriate representative population sample to determine the actual burden of viral hepatitis in the general population is often a challenge, due to the high cost of participants' recruitment and the indifference people demonstrate to such scientific surveys especially in the developing world [7]. Using data on prospective blood donors as a proxy, this study is therefore aimed at estimating the prevalence and the five-year (2012–2016) trend of viral hepatitis (HBV and HCV) infection among the apparently healthy asymptomatic adult populations in the Ho Municipality, Ghana.

## 2. Methods

### 2.1. Study Design and Study Site

The study was a retrospective analysis of secondary data of prospective blood donors who visited the Ho Municipal Hospital from January 2012 to December 2016. The study included 4,180 male and female donors aged between 18 and 58 years whose complete records were available for review. Demographic parameters included age, sex, and place of residence. Screening results of infectious markers (HBV and HCV) using rapid diagnostic test kits were obtained from the archives at the blood bank.

### 2.2. Study Area

Ho Municipality is one of the five municipalities in the Volta Region established by a Legislative Instrument (LI) 2074 of 2012. The municipality has Ho as its capital which serves as the capital and economic hub of the Volta Region. The Municipality is located between latitudes 6°20′′N and 6°55′′N and longitudes 0°12′E and 0°53′E. It shares boundaries with Adaklu and Agotime-Ziope Districts to the South, Ho West District to the North and West, and the Republic of Togo to the East. It has a total land size of 2,361 square kilometres thus representing 11.5 percent of the region's total land area [8]. The municipality has a total population of 192,871 with 94,951 males and 97,920 females and a growth rate of 1.17% as of the year 2010 [9].

### 2.3. Data Analysis

Data collected were entered into Microsoft Excel 2013 spreadsheet and validated for entry errors. Data was presented as frequencies and proportions. Differences between proportions and trends analysis were done using Fisher's exact test and chi square test for trend where appropriate. A *p* value < 0.05 was considered as statistically significant. IBM Statistical Package for the Social Sciences (SPSS Inc., Chicago, USA; (http://www.spss.com)) version 22.00 was used for data analysis.

### 2.4. Ethical Consideration

Written approval for the study was sought and obtained from the management of Ho Municipal Hospital. Ethical clearance for the study was granted by the Ethical Review and Scientific Committee of the School of Allied Health Sciences, University of Health and Allied Sciences, Ho* (UHAS-SAHS-ERSC: 029A/2017)*. Analysis of the data was anonymous and nonlinked, and no donor names were retrieved from the archives.

## 3. Results 

A total of 4,180 prospective blood donors were recorded for the five-year period under review at the Ho Municipal Hospital. Seventy-seven (1.84%) out of the total potential donor population tested positive for HCV antibody, while 290 (6.94%) were seropositive for hepatitis B virus surface antigenaemia. From the five-year period under review, HBsAg expression remained steady throughout the period (*p for trend* = 0.4592) whereas HCV antibody reactivity recorded a significant year-on-year decreasing trend from 2012 up to 2015 with a percentage reduction from 2.71 to 0.93 (*p for trend* = 0.0161) (see Table 1).

A total of three thousand six hundred and ninety-six (3696) prospective male blood donors were recorded at the Ho Municipal Hospital blood bank for the five-year period under review. Among these, 250 (6.8%) were positive for HBsAg while 53 (1.4%) tested positive for HCV antibody. The seropositivity of HBsAg year-on-year trend was observed to be statistically steady during the period under review; however the observed peak year was 2012 (7.47%) and the trough was observed in the year 2016 (5.7%). As seen from Table 2, though not statistically significant the lowest seropositivity of HCV among the male population was seen in 2015, but the rate inched up in the subsequent year.

Among the four hundred and eighty-four (484) prospective female blood donors that were recorded for the five-year review, 40 (8.3%) were seropositive for HBsAg whereas 24 (5.0%) tested positive for HCV. The review observed a steady HBsAg seropositivity throughout the period. However, a statistically significant year-on-year decreasing trend of incidence of HCV infection was observed (*p* for trend 0.0055). As seen from Table 3, HCV seropositivity declined from 11.0% in 2012 to 1.9% in 2016.

Majority of the prospective blood donors were within the age bracket of 20–39 years (82.04%). Significant age variation in HBV antigenaemia was seen. Hepatitis B viral seropositivity peaked among the younger population 11.24% (less than 20 years group) and troughed among the older population 0.92% (Above 50 years group). Hepatitis C viral antibody reactivity showed no significant differential age distribution (see Table 4).

**Figure 1:**
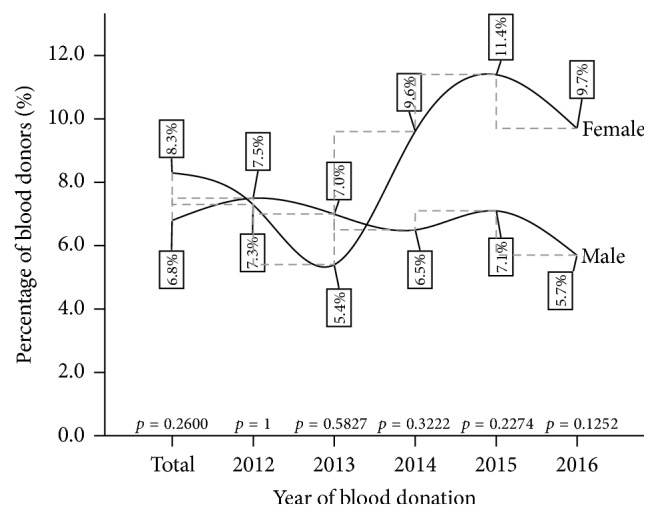
A five-year gender variation of HBsAg infection among blood donors at Ho Municipal Hospital.

Even though no significant gender variation in HBsAg was observed for both the cumulative infection burden (*p* = 0.2600) and the year-on-year comparisons throughout the review period, the pattern of HBsAg antigenaemia saw a greater female HBsAg prevalence rate in the last three years of the period under review. There were a tie in 2012 and a reduction in male prevalence from 7.1% to 5.7% in the last two final years (see Figure 1).

**Figure 2:**
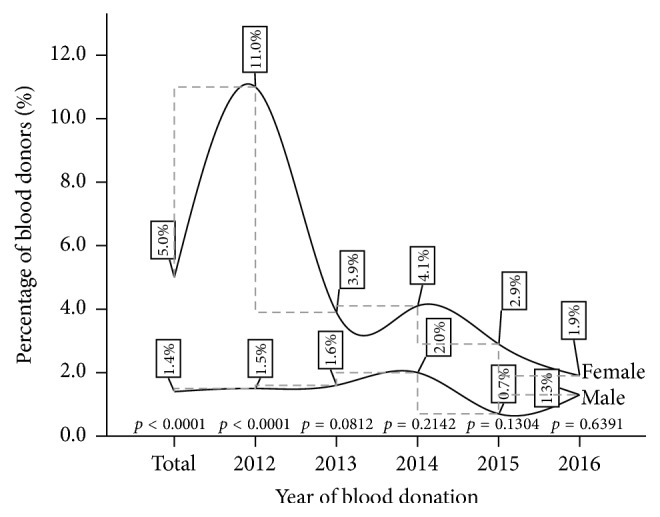
A five-year gender variation of HCV infection among blood donors at Ho Municipal Hospital.

The cumulative percentage of HCV antibody burden was found to be significantly higher among the female population (5.0%) compared to their male counterparts (1.4%) (*p* < 0.0001). In the first year of the review (2012), significant difference in HCV infectious burden tilted toward the female donor population (*p* < 0.0001); this gap became narrowed ending with the lowest prevalence for the females (see Figure 2).

## 4. Discussion

Viral infections remain a major cause of morbidity and mortality in the developing world [10]. In the current study, a total of 4,180 Ghanaian asymptomatic adult population were screened for the five-year period under review (2012–2016) at the Ho Municipal Hospital. Out of this number, 290 (6.94%) were seropositive for HBV surface antigenaemia while 77 (1.84%) tested positive for HCV antibody. Among the male population, 250 (6.8%) were seropositive for HBsAg whereas 53 (1.4%) tested positive for HCV antibody compared to the 40 (8.3%) and 24 (5.0%) that reacted to HBsAg and HCV antibody, respectively, in the female population.

The results suggest that the burden of viral infections among the study participants falls within the category of intermediate to high endemicity. Prior to this work, results of similar magnitude have been reported among Ghanaians. A recent work in Koforidua undertaken by Alomatu [11] reported a prevalence of 13.2% and 8% for HBsAg and HCV, respectively. In their study at Kintampo, Walana et al. [12] reported a seroprevalence of 9.6% for HBV and 4.4% for HCV infections among asymptomatic adult population. In Agogo, a study by Nkrumah et al. [13] reported an overall prevalence of 13.8% and 9.4%, respectively, for HBV and HCV among a rural community. In other Sub-Saharan countries, results comparable to that of the current finding have been reported in Cameroon [14], Togo [15], Nigeria [16, 17], Ethiopia [18, 19], and Kenya [20]. In contrast, low endemicity of viral infections have been shown to occur in Southeastern Nigeria (0.3%, 0.2%) [21], respectively, for HBV and HCV infections.

Our study revealed a higher burden of viral hepatitis infections among females in comparison to their male counterparts. This finding is partly in agreement with that of Nkrumah et al. [13] where femininity was highly associated with HBV infections but not HCV infections which was higher in males. In contrast, Alomatu [11] and Walana et al. [12] in their studies observed a higher prevalence of viral hepatitis (HBV and HCV) among males compared to their female counterparts. It is not quite clear from the present study what exactly could account for the female preponderance to viral infectivity. Postulated factors to female higher predisposition to acquiring sexually transmitted infections than men include early onset of sexual activity, wider surface of the vagina, longer semen-vaginal contact, lower level of education, iatrogenic acquisition in health facilities where women are more often admitted than men, especially during pregnancy and at delivery, and occupation as well as lower standard of living [22–24].

The decreased risk of blood-borne pathogens in developed countries is attributable to the success in preventing established infectious agents from entering the blood circulation; however, the same may not be entirely true for the developing world [25]. In the current study, though the year-on-year trend analysis of infectious markers revealed a significant decreasing tendency of HCV infections in the general donor population and the female donor population (Tables 1 and 3), HBV seroantigenaemia remained largely steady throughout the period under review (Tables 1, 2, and 3).

Age is thought to be strongly associated with the transmission of infectious markers, with the younger age group of less than 30 years shown to be significantly susceptible [19]. In the present study, a significant age variation in HBV antigenaemia was observed. Hepatitis B viral seropositivity peaked among the younger population (less than 20 years' group) at 11.24% and troughed among the older population (above 50 years group) at 0.92%. (Table 4). The finding is similar to those reported previously where viral hepatitis was associated with the younger population [11]. The younger generation of the study participants with a higher viral infectious burden could have socioeconomic implications. These individuals may be of school going age and could be chronically living with the infection for a considerable length of time. Also, when left untreated, the infection could advance thereby leading to a possible major organ failure, especially the liver. Moreover, because they are asymptomatic, they could be a potential source of spread of these infections in the general healthy population.

## 5. Conclusion

The prevalence of viral infections among asymptomatic adult population in the Ho Municipality is estimated at the intermediate to high endemicity level. The burden of HCV significantly varied over the period under review, but the rate of HBV infection remained steady throughout the period. Preventive measures to reduce the burden are urgently needed and should be targeted at the younger generation.

## Figures and Tables

**Table 1:** Year-on-year trends of hepatitis viral infectious markers among prospective blood donors at the Ho Municipal Hospital.

Parameter	Total	2012	2013	2014	2015	2016	*p* for trends
Blood donors	4180	884	1128	630	646	892	Nd
*HBsAg*	290 (6.94)	66 (7.47)	77 (6.83)	43 (6.83)	49 (7.59)	55 (6.17)	0.4592
*HCV*	77 (1.84)	24 (2.71)	21 (1.86)	14 (2.22)	6 (0.93)	12 (1.35)	0.0161

Data is presented as figure with corresponding percentages in parenthesis. HBsAg: hepatitis B surface antigen. Anti-HCV: hepatitis C virus antibody. *p* is significant at 0.05. nd: statistical trend analysis not done.

**Table 2:** Year-on-year trends of hepatitis viral infectious markers among prospective male blood donors at the Ho Municipal Hospital.

Parameters	Total	2012	2013	2014	2015	2016	*p* for trends
Donors	3696	776	999	560	572	789	Nd
HBsAg	250 (6.76)	58 (7.47)	70 (7.00)	36 (6.42)	41 (7.16)	45 (5.70)	0.2019
Anti-HCV	53 (1.43)	12 (1.54)	16 (1.60)	11 (2.00)	4 (0.99)	10 (1.26)	0.3246

Data is presented as figure with corresponding percentages in parenthesis. HBsAg: hepatitis B surface antigen. Anti-HCV: hepatitis C virus antibody. *p* is significant at 0.05. nd: statistical trend analysis not done.

**Table 3:** Year-on-year trend of infectious markers among prospective female blood donors at the Ho Municipal Hospital.

Parameters	Total	2012	2013	2014	2015	2016	*p* for trends
Donors	484	108	129	70	74	103	Nd
HBsAg	40 (8.26)	8 (7.40)	7 (5.42)	7 (10.00)	8 (10.81)	10 (9.70)	0.2207
Anti-HCV	24 (4.95)	12 (11.11)	5 (3.87)	3 (4.1)	2 (2.70)	2 (1.94)	0.0055

Data is presented as figure with corresponding percentages in parenthesis. HBsAg: hepatitis B surface antigen. Anti-HCV: hepatitis C virus antibody. *p* is significant at 0.05. nd: statistical trend analysis not done.

**Table 4:** Age variation of the infectious markers among prospective blood donors at the Ho Municipal Hospital.

Parameter	Donors (*n*—4180)	HBsAg (*n*—290)	Anti-HCV (*n*—77)
<20	178 (4.26)	20 (11.24)	2 (1.12)
20–29	2321 (55.53)	157 (6.76)	44 (1.90)
30–39	1108 (26.51)	83 (7.49)	17 (1.53)
40–49	464 (11.10)	29 (6.25)	12 (2.59)
≥50	109 (2.61)	1 (0.92)	2 (1.83)
*p* *value*	*Nd*	*0.016*	*0.5176*

Data is presented as figures with corresponding percentages in parenthesis; HBsAg: hepatitis B surface antigen. Anti-HCV: hepatitis C virus antibody. *p* is significant at 0.05. nd: statistical analysis for difference not done.
